# Wearable and Portable Electrocardiographic Devices as Modern Cardiac Telemetry Solutions in Pediatrics: A Systematic Review

**DOI:** 10.3390/jcm15082883

**Published:** 2026-04-10

**Authors:** Magdalena Warych, Jakub Zabłocki, Julia Krawczyk, Jan Herc, Piotr Wieniawski, Radosław Pietrzak

**Affiliations:** Department of Pediatric Cardiology and General Pediatrics, Medical University of Warsaw, 02-091 Warsaw, Poland

**Keywords:** portable electrocardiograph, smartwatch, ECG, KardiaMobile, arrhythmia in children, event recorder

## Abstract

**Background/Objectives**: Portable and wearable ECG technologies are increasingly used in adult cardiac monitoring. However, evidence supporting their feasibility and diagnostic performance in pediatric populations remains limited. This systematic review evaluates the diagnostic accuracy, usability, artifact susceptibility, and user acceptance of mobile ECG technologies in pediatric cardiology. **Methods**: A systematic literature search was performed in the Embase, PubMed, Scopus, and Web of Science databases. The review was conducted in accordance with the PRISMA 2020 guidelines and was registered in the PROSPERO database. **Results**: A total of 30 publications were included in the final analysis. Portable ECG devices demonstrated good feasibility diagnostic utility in children. Handheld systems provided high-quality tracings with strong agreement with standard 12-lead ECGs and higher adherence, as well as user satisfaction compared with conventional event recorders. However, automated rhythm classification frequently misidentified pediatric arrhythmias. Smartwatch-based ECG recordings showed high diagnostic accuracy when manually interpreted, but automated algorithms were unreliable, particularly for tachyarrhythmias and conduction abnormalities. Alternative electrode placement strategies improved smartwatch performance, and patient acceptance was consistently high. ECG patch monitoring, particularly with extended-wear devices, achieved the highest diagnostic yield, detecting arrhythmias often missed by short-duration Holter monitoring while maintaining comparable signal quality. **Conclusions**: Mobile ECG technologies represent a promising adjunct for pediatric rhythm surveillance, offering diagnostic performance comparable to standard modalities when interpreted by clinicians and improved usability and patient acceptance. Persistent limitations include the poor reliability of adult-oriented automated algorithms and the underrepresentation of younger children and the predominantly off-label use of these devices in pediatric populations, underscoring the need for pediatric-specific algorithm development and age-adapted device design.

## 1. Introduction

In recent years, the healthcare sector has undergone a profound transformation, driven by advances in digital health technologies and the adoption of telemedicine, which have revolutionized not only clinical diagnostics and therapeutic interventions but also patient monitoring, both in hospital settings and beyond. Particularly in ambulatory care, the use of telemedical solutions serves as a strategy to address disparities in healthcare access, especially among populations in rural or underserved regions [1]. By enabling long-term monitoring and real-time feedback, these technologies facilitate early detection of pathological changes and reduce the time required to access professional medical support [2].

The field of cardiology stands at the forefront of this digital transformation. Among the available tools are devices capable of capturing and analyzing electrocardiographic (ECG) data in real time. These devices—both wearable and portable—enable continuous rhythm surveillance, arrhythmia detection, and assessment of cardiac function [3]. Their clinical applications are broad, ranging from atrial fibrillation screening [4] and heart rate variability analysis [5] to post-cardiac event monitoring [6] and chronic heart failure management [7].

A growing number of wearable and portable ECG devices have entered the market in recent years. For the purposes of this review, they have been categorized into three subgroups: (1) handheld ECG recorders, (2) smartwatches (SW), and (3) adhesive ECG patches. The most widely studied examples of these devices include systems such as KardiaMobile (KM) for handheld devices, Apple Watch (AW) for SW, and Zio XT patch for adhesive ECG patches. They differ in signal quality, lead configuration, usability, and integration with mobile health platforms. While many are validated for adult use, their performance and appropriateness in children remain less established.

Handheld ECG devices represent portable single- or multi-lead recorders that are typically activated by the patient during symptoms. KM is a widely used smartphone-based ECG system available in both single-lead (KM1L) and six-lead (KM6L) configurations. To record an ECG tracing, the patient places the fingers of both hands on two small metal electrodes and maintains contact for 30 s. In KM6L, an additional third electrode touches the skin of the lower extremity, completing the electrical circuit that enables the generation of six standard limb leads. The recording is transmitted to the patient’s smartphone via Bluetooth in real time. The dedicated Kardia app stores tracings, allows them to be exported as a PDF, and enables patients to share results with their physician immediately. Notably, the procedure can be performed entirely outside of a clinical setting, without direct supervision by healthcare personnel. The KM system incorporates automatic algorithms validated in adults that can detect, among others, sinus rhythm, atrial fibrillation (AF), bradycardia, and tachycardia. In adult populations, KM devices are now commonly used for ambulatory cardiac monitoring. Their diagnostic accuracy compared with standard 12-lead ECG (12LECG) has been confirmed in multiple clinical trials [8,9]. Reflecting this, devices such as KM are already mentioned in the 2024 ESC Guidelines as valid screening tools for the detection and diagnosis of AF [10]. On the other hand, the manufacturer states that the device “has not been tested and is not intended for pediatric use” [11]. In consequence, the KM automated algorithms are not adapted for children and do not allow the user’s age to be set below 18 years. Zenicor ECG device is another type of device from the handheld ECG group. Although it is substantially less popular and the literature data is substantially more scarce, the manufacturer declares the effectiveness and ease-of-use of this system in children [12].

SWs constitute the most prevalent group of wearables in contemporary mobile ECG devices. They enable obtaining a single-lead tracing, usually by utilizing two sensors—one at the bottom of the watch and the other on the crown of the watch. The use of smartwatches in arrhythmic patients has been evaluated in multiple studies, including research by Perez et al. (2019), which assessed the efficacy of the AW to detect AF in a group of over 400,000 participants and reported a positive predictive value of 0.87 [13]. However, official statements from the largest manufacturers of smartwatches confirm that the ECG feature is available only for people aged 22 or older [14].

Zio XT Patch is a small, wireless device designed specifically for heart rhythm and arrhythmia monitoring. It continuously records a single-lead ECG for up to 14 days and allows patients to press an event button to mark symptom onset. This enables physicians to assess whether the symptoms correlate with an arrhythmic event. The patch is adhered directly to the chest. It can be worn during normal daily activities (including showering, exercising, and sleeping), which makes the monitoring experience more convenient and less restrictive than conventional Holter monitoring. Nonetheless, according to the Zio XT Clinical Reference Manual, the “safety and effectiveness of the Zio XT Patch on pediatric patients (<18 years) has not been established”. [15].

As mentioned above, a variety of non-hospital-based ECG monitoring methods are available on the market. However, a notable gap exists in the literature: most studies are focused on adult populations, where cardiovascular conditions are more prevalent. In the pediatric population, arrhythmias are predominantly associated with congenital conditions, often associated with structural heart disease, post-surgical complications, or occurring idiopathically [16]. Their estimated incidence is approximately 24.4 per 100,000 live births in the United Kingdom [17]. The use of mobile devices may serve an even more critical role in children than in adults [18]. Children—particularly infants and toddlers—are often unable to effectively communicate symptoms such as palpitations, chest pain, or syncope. For parents of children with congenital or acquired heart defects, even subtle clinical signs can be alarming. In these cases, immediate and remote cardiac monitoring, supported by healthcare professionals, can provide significant reassurance, enable timely intervention, and potentially improve clinical outcomes.

In this systematic review, we examine the current state of knowledge about the use of portable and wearable ECG-monitoring devices in pediatric cardiology.

We predominantly aimed to answer the following questions:

1. How accurate are those devices in arrhythmia detection and rhythm identification?
2. How feasible are those devices for ECG interval measurements and heart rate (HR) identification?
3. How do artifacts and external factors affect the assessment of the tracings obtained with those devices?
4. How high is the patient satisfaction with the use of those devices?

## 2. Materials and Methods

The systematic review of available literature was conducted in accordance with the PRISMA (Preferred Reporting Items for Systematic reviews and Meta-analyses) 2020 guidelines [19]. The study protocol has been registered in the PROSPERO database with a registration number CRD420251056211. Original clinical research regarding the application of either smartwatches, handheld ECG devices, or ECG patches was included and underwent full-text assessment. Systematic reviews, meta-analyses, case reports, conference abstracts, letters, protocols, and errata were excluded. The inclusion criteria required that the studied population consisted of pediatric patients (<18 years of age), with or without congenital heart disease (CHD) or other comorbidities, and included both in-patient and outpatient settings, regardless of the clinical context. A comprehensive search of the following databases was conducted on 3 March 2026: Embase, PubMed, Scopus, and Web of Science. The following query was applied: (“wearable ECG device” OR “single-lead ECG” OR “handheld ECG” OR “mobile ECG” OR “wearable electrocardiography” OR “KardiaMobile” OR “AliveCor” OR “Zio patch” OR “ECG patch” OR “smartwatch” OR “Apple Watch”) AND ECG AND (“pediatric” OR “children”), resulting in a total of 407 records extracted for screening. MW and JZ independently conducted the screening and the risk of bias assessments and extracted the data from each ultimately included record. The PRISMA 2020 checklist has been included in the Supplementary Table S1. 

The following data were extracted from the research: authors, publication year, DOI, enrollment period, study population size and characteristics, ECG devices used, and outcomes.

**Figure 1:**
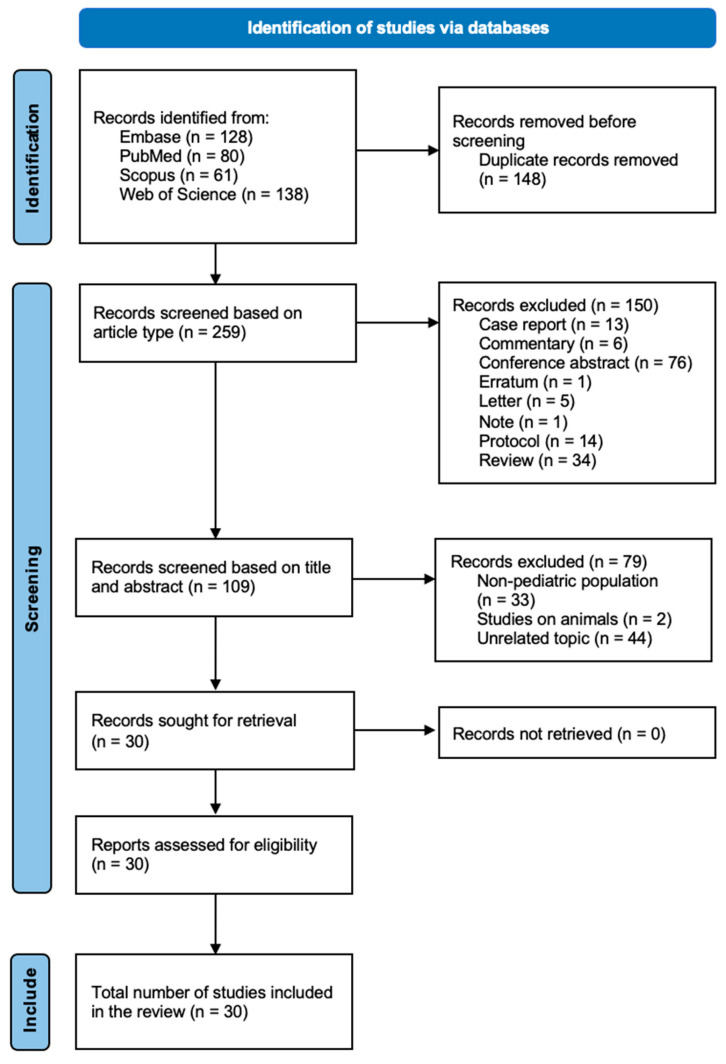
PRISMA flowchart.

At the first screening stage, 148 duplicates were removed. Then, 259 records were screened for article type using the criteria above. A total of 109 records were assessed based on their titles and abstracts. The reasons for removal at this stage included: (a) the study population not being pediatric (*n* = 33), (b) studies on animals (*n* = 2), (c) an unrelated topic of the study (*n* = 44). Full texts were sought for 30 records and all were successfully retrieved. Those research articles were assessed for eligibility, and all met the inclusion criteria (Figure 1).

Risk of bias was assessed using one of three validated tools, selected according to the methodological design of each study. For diagnostic accuracy studies, we employed the QUADAS-2 (Quality Assessment of Diagnostic Accuracy Studies) instrument [20] (Supplementary Table S2). Within QUADAS-2, both risk of bias and applicability concerns were evaluated across all domains and classified as low, unclear, or high. In nonrandomized cohort studies, when QUADAS-2 was not applicable, the Newcastle-Ottawa Scale [21] was used. For this review, the following risk-of-bias cut-offs were categorized with the following thresholds: 7–9 points—low risk of bias; 4–6 points—moderate risk of bias; 0–3 points—high risk of bias (Supplementary Table S3). Randomized controlled trials were evaluated with the Cochrane RoB 2 tool [22] (Supplementary Table S4).

The remaining 30 studies were divided into three groups and were further analyzed based on the device: (1) KM, (2) SW, and (3) Adhesive ECG Patches.

## 3. Results

### 3.1. Handheld ECG Devices

A total of 12 studies evaluated the clinical utility and usability of handheld ECG devices in the pediatric population [23,24,25,26,27,28,29,30,31,32,33,34] (Table 1). Most studies investigated the use of KM (either KM1L or KM6L) in children, while one study focused on Zenicor-ECG handheld system. These investigations ranged from assessing the technical quality of tracings and the feasibility of arrhythmia detection to comparing them with conventional 12-/15-lead ECGs or traditional event monitors and evaluating the accuracy of specific electrocardiographic measurements.

Across studies evaluating handheld ECG devices in pediatric populations, the proportion of diagnostically interpretable recordings ranged from 90–96% [28,34] indicating generally high feasibility of home-based ECG acquisition. Diagnostic accuracy for arrhythmia identification varied depending on recording configuration and clinical context, with reported values ranging from 59% to 76% [26] for differentiation of SVT mechanisms. Agreement with standard ECG for HR measurement was strong, with correlation ranging from r = 0.44 to r = 0.92, while agreement for QRS axis was high (correlation up to r = 0.96) [30,31]. QT and QTc measurements varied from r = 0.47 to r = 0.83 [28,29,30] and sensitivity for detecting prolonged QTc of 54–80% [27], although specificity remained high at 95–96% [27]. Significant artifact rates varied considerably depending on device configuration and recording conditions, ranging from 7% to 35% [27,29,31], with higher rates reported in younger children and unsupervised recordings.

#### 3.1.1. Arrhythmia Detection and Rhythm Classification

One of the earliest pediatric studies evaluating handheld ECG devices was conducted by Nguyen et al. (2015) [34], who provided KM1L to 35 patients with documented arrhythmias, including AF, supraventricular tachycardia (SVT), atrial tachycardia (AT), and ventricular tachycardia (VT). Ninety-six percent of remotely transmitted home-based recordings were of diagnostic quality, enabling clinicians to identify SVT and AF reliably. Significantly, the availability of these recordings directly influenced clinical management, prompting outpatient interventions in three patients. Given the small sample size, further research was required. The topic of arrhythmia detection was simultaneously addressed by Ferdman et al. (2015) [26], who investigated KM1L’s ability to discriminate between atrioventricular reentrant tachycardia (AVRT) and atrioventricular nodal reentrant tachycardia (AVNRT) during electrophysiology-guided ablation procedures, which served as the gold-standard reference. Diagnostic accuracy ranged from 59% to 76% depending on device position. During the recordings, KM1L was placed on the patient’s upper left chest in three positions—horizontal and 60° rotation were significantly more diagnostic than 120° for detecting SVT.

While these early studies were limited by small sample sizes, they provided initial evidence supporting the feasibility of KM in pediatric rhythm evaluation. It was the foundation for subsequent, more robust validation studies comparing KM with the established clinical gold standard: 12- or 15-lead ECGs. Kurath-Koller et al. (2026) [30] significantly expanded this evidence base by demonstrating that KM could provide high-quality recording and reliable rhythm documentation during symptoms in 69% of cases, effectively identifying or excluding significant arrhythmias in children.

Furthermore, Dahlqvist et al. (2014) [25] extended the application of handheld devices to complex congenital heart disease (CHD). They evaluated the Zenicor-ECG handheld system in 27 children with Fontan circulation over a 14-day period. The device successfully identified a previously undiagnosed SVT in a symptomatic patient, undetected by prior 24-h Holter monitoring, as well as ventricular arrhythmias in an asymptomatic patient.

#### 3.1.2. Accuracy of Interval and Heart Rate Measurements

Several studies assessed the accuracy of portable ECG devices in measuring HR and ECG intervals, including QT and corrected QT interval (QTc), QRS duration (QRSd), and PR.

For HR and general interval measurements, correlations were robust. Karacan et al. (2019) [29] reported a strong correlation for mean HR (r = 0.92). On the other hand, Kurath-Koller et al. (2026) [30] demonstrated moderate correlation between KM and the gold standard in terms of HR (r = 0.44), attributed to the non-simultaneous nature of recordings and inherent pediatric heart rate variability. Despite this, they found a near-perfect correlation for the QRS axis (r-0.96), reinforcing the device’s diagnostic accuracy. Gropler et al. (2018) [28] observed r > 0.8 for both automated and manual HR assessment, and similarly strong correlations for PR and QRSd. Girvin et al. (2023) [27] confirmed minimal bias for QRSd (+2 ms) and near-perfect axis agreement (bias 0.5°) between KM6L and 15-lead ECG. Similarly, Lawley et al. (2024) [31] reported excellent agreement for QRSd (ICC = 0.911) and QRS axis (ICC = 0.962). Miller et al. (2024) [33] investigated the clinical usability of KM6L in children following ablation procedures. Electrophysiologists rated KM tracings as high-quality and clinically useful alternatives to 12LECG.

In contrast, QT and QTc screening results showed some significant discrepancies. Karacan et al. (2019) [29] found a strong correlation for QT (r = 0.83), but only a moderate correlation for QTc (r = 0.57). It was attributed to heart rate variability affecting the Bazett correction. Gropler et al. (2018) [28] reported a mean ΔQTc of 15.6 ± 12.7 ms (r = 0.83) in KM1L compared with 12LECG, with 76% of values within ±20 ms. For prolonged QTc, sensitivity and specificity were 80% and 96%, respectively. Girvin et al. (2023) [27] found a negligible QTc bias (−0.6 ms) in KM6L. They reported a sensitivity of 54% and a specificity of 95% for prolonged QTc, suggesting slightly reduced sensitivity of KM6L versus KM1L for abnormal QTc. Lawley et al. (2024) [31] demonstrated strong reproducibility (ICC ≥ 0.85) and substantial agreement for prolonged QTc classification (κ = 0.714, *p* < 0.001).

A limitation noted in Gropler et al. (2018) [28] was the imperfect performance of automated rhythm interpretation in cases with interval abnormalities. The authors reported four false-positive “possible AF” readings, all later identified as benign variants (including sinus tachycardia or aberrantly conducted premature atrial contractions on expert review. Moreover, Miller et al. (2024) [33] noted that the device may be less suitable for detecting subtle pre-excitation patterns. This finding highlights the importance of manual over-read by clinicians, particularly when interpreting tracings with borderline interval values or atypical morphology.

#### 3.1.3. Influence of Artifacts and Technical Factors

Artifacts were present in 7–35% of recordings, depending on device type and recording conditions. According to studies evaluating the KM6L configuration—Girvin et al. (2023) [27], Lawley et al. (2024) [31], artifacts occurred more frequently than with the KM1L. In the first study, which included participants aged 6–18 years, the authors reported an artifact rate of 35%. They noted that smaller body size and difficulty maintaining leg contact were limiting factors in younger children. The artifact rate decreased with age, with the highest success rate observed in children aged ≥12 years. In the second study, with a population age range of 7–17 years, suspected lead misplacement was identified in 18% of unsupervised home recordings. Families of younger children more often required assistance, yet home use remained feasible across the entire cohort.

The KM1L device appeared to produce fewer artifacts in pediatric users than the 6-lead version, likely due to its simpler design and the absence of the lower-limb lead. Gropler et al. (2018) [28] reported that 10% of tracings required repeat acquisition due to motion or poor electrode contact, with the youngest patients (infants and toddlers) needing caregiver assistance and showing lower diagnostic quality on the first attempt. Similarly, Karacan et al. (2019) [29] excluded 7% of recordings due to motion noise or unclear T-wave termination, findings most common in children under 6 years of age. In contrast, Miller et al. (2024) [33] reported no significant artifact issues. However, all recordings in that study were obtained under direct supervision by trained providers.

A valid perspective was raised by Kurath-Koller et al. (2026) [30] who noted that parameters known to have lower signal amplitudes or higher susceptibility to artifacts (e.g., P wave amplitude, PR interval, QRS duration or QTc) are prone to lower interobserver agreement compared to other parameters. Moreover, the authors described two patients initially unable to undergo KM6L recoding due to remains of talcum powder from prior climbing exercise which impaired signal transmission through device’s electrodes.

In conclusion, artifacts were more common in younger children and in unsupervised settings. Still, even in these conditions, most tracings remained of diagnostic quality, supporting the feasibility of home-based ECG acquisition with KM devices.

#### 3.1.4. Performance in Patients with Implanted Pacemakers

Only two of the analyzed studies provided detailed technical evaluation regarding pediatric patients with pacemakers. Gropler et al. (2018) [28] included three patients with pacemaker rhythm in the interval validation cohort. KM1L accurately recorded pacing spikes and QRS morphology, with pacing rhythms correctly interpreted by physicians but occasionally misclassified as AF by the device’s automated algorithm. Karacan et al. (2019) [29] also included 17 children with conduction abnormalities, among them pacemaker recipients. KM1L signals were fully interpretable, with no loss of pacing artifact or signal distortion, leading the authors to conclude that the device can be safely used in children with implanted pacemakers for rhythm documentation.

Taken together, the available data, though limited to a small sample, indicate that KM appears to perform equally well in patients with paced rhythms as in those with intrinsic cardiac activity. Larger, dedicated studies are needed to confirm these findings in the pediatric population. However, based on adult data, the device does not appear to generate significant artifacts or signal interference in the presence of pacing stimuli, suggesting overall compatibility with implanted cardiac devices [35].

#### 3.1.5. Comparison with Traditional Devices

Three studies compared KM with traditional event recorders such as Cardiocall or MicroER. Conventional devices rely on adhesive chest electrodes and require the patient to press a button during symptoms, whereas KM’s wireless, fingertip-activated design does not.

Macinnes et al. (2019) [32] reported that tachyarrhythmia was documented in 25% of KM users versus only 6% with Cardiocall (*p* < 0.001), while the proportion of non-interpretable tracings was similar between groups (8%). Notably, 38% of patients in the conventional group found it difficult to upload or send ECGs, compared with only 5% in the KM group, resulting in 98% of KM users submitting at least one recording, versus 69% in the conventional group (*p* < 0.05). Al-mousily et al. (2021) [24] found a similar overall diagnostic yield between KM and MicroER, but total ECG transmissions were significantly higher with KM (692 vs. 142; *p* < 0.001), despite comparable population sizes across device cohorts. Al Riyami et al. (2025) [23] reported that rhythm was documented during palpitations in 51% of KM users versus 44% of Cardiocall users, a difference that was not statistically significant. However, a significantly greater proportion of KM patients transmitted at least one recording (70% vs. 49%, *p* = 0.037). Survey response rates were low overall, but higher among KM users, who also reported greater satisfaction (83% vs. 58%).

Moreover, Kurath-Koller et al. (2026) [30] compared KM6L with standard 12LECG and the AW. Although the correlations for most intervals were low-to-moderate for most ECG intervals in KM, this may be due to time span between the KM and 12LECG recording—the latter was conducted within 30 min from the handheld device recording.

Collectively, these studies demonstrate that KM provides at least equivalent—and often superior—diagnostic yield compared with traditional event recorders, while offering a markedly better patient experience. In all studies that assessed comfort and acceptance, KM was consistently preferred, primarily because of the absence of adhesive electrodes, the intuitive mobile interface, and the ease of recording.

#### 3.1.6. Patient Satisfaction

Several studies evaluated patient and family experience with handheld ECG devices, including usability, adherence, and overall satisfaction. Lawley et al. (2024) [31] reported overall positive acceptance of KM6L, with families considering the device easy to use in both supervised and unsupervised settings. Miller et al. (2024) [33] found that 100% of patients reported KM was easy to use, 92% reported the app was easy to use, and 96% felt comfortable using the device without a healthcare professional present. The mean age in this study was 13.2 (SD = 4). Patients were mostly old enough to perform the procedure by themselves, only with adult supervision.

The feasibility and high acceptance of handheld ECGs were also confirmed in other research. Kurath-Koller et al. (2026) [30] demonstrated that KM was perceived as highly user-friendly, requiring significantly less time for a successful recording (median = 5 min) compared to the AW (median = 15 min), and comparable to standard ECG (median = 4 min), which directly translated to better cooperation with children as young as 4 years. Similarly, Dahlqvist et al. (2014) [25] reported excellent adherence in a 14-day monitoring protocol among children with Fontan circulation. The patients successfully integrated the Zenicor-ECG monitor into their daily routines, including school and physical activities, performing an average of 27 recordings per person without significant discomfort or barriers.

In conclusion, these findings highlight the high acceptability of KM devices among pediatric patients and their families. Future studies focusing on toddlers and preschool children are needed to determine whether the independent use of handheld ECG devices is feasible in this age group.

### 3.2. Smartwatches

A total of 12 studies investigated the feasibility, accuracy, and clinical applicability of smartwatch-derived electrocardiograms (SW-ECGs) in pediatric populations [30,36,37,38,39,40,41,42,43,44,45,46,47] [Table 2]. These studies primarily evaluated the AW, with some including the Withings ScanWatch and other devices, such as the Garmin Vivoactive 4S and Fitbit Sense, across a variety of clinical settings, ranging from routine screenings to electrophysiological studies. Collectively, these data provide a comprehensive overview of the advantages and limitations of SW-ECGs in children.

Studies evaluating SW-ECG systems in pediatric populations demonstrated a high proportion of interpretable tracings, generally ranging from 94.9% [38] to 100% [43]. HR measurement showed excellent agreement with reference ECG (r = 0.88–0.99) [37,46]. However, automated rhythm classification algorithms showed variable diagnostic performance, with reported sensitivity ranging from 38.9% [45] to 96% [42] and specificity from 76.7% [45] to 100% [39]. A notable limitation was the high proportion of inconclusive automated classifications, reported in 23% [41]. to 38% [38] of recordings. Artifact rates were generally lower than those observed in handheld devices, typically ranging from 0.9% [39] to 19% [41], although motion and poor electrode contact remained important technical limitations [37].

#### 3.2.1. Arrhythmia Detection and Rhythm Classification

Across the included studies, rhythm classification based on SW-ECG recordings showed high diagnostic concordance with standard 12LECG when interpreted manually by clinicians, whereas automated algorithms consistently demonstrated lower performance.

Manual physician over-read achieved 100% diagnostic concordance with 12LECG [41] and excellent agreement in classifying tracings either normal or abnormal [39]; κ = 0.91. In contrast, automated algorithms demonstrated significant underperformance. Kobel et al. (2022) [38] reported that the native AW algorithm correctly classified only approximately 50% of sinus rhythms, labeling up to 38% as “inconclusive”. Similarly, Littell et al. [41] found that the automated algorithm achieved a specificity of only 75%, classifying 23% of cases as “inconclusive” and 1.6% as “incorrect” (false positives, e.g., reporting AF in normal sinus rhythm) and failure to detect subtle conduction abnormalities, e.g., pre-excitation, junctional rhythm, or intraventricular conduction delay.

Furthermore, automated algorithms frequently miss clinically significant findings that are apparent upon manual review. Littell et al. (2022) [41] found that in 9.8% of cases, additional abnormalities (e.g., pre-excitation, junctional rhythm, or intraventricular conduction delay) were identified by the over-read despite the algorithm classifying the rhythm as “accurate with missed findings”.

Nash et al. (2024) [42] further highlighted that AW performance in distinguishing sinus from pathological rhythms remained high at rest (89–96%), but deteriorated significantly during tachyarrhythmias, illustrating a clinically relevant limitation for real-time automated detection.

More recently, artificial intelligence-based approaches have been explored to improve automated rhythm interpretation. An interesting perspective arises from research by Teich et al. (2023) [45]. In this study, artificial intelligence-based approaches for assessing AW-derived ECG demonstrated improved sensitivity (66.7%) and specificity (96.7%) for rhythm classification compared with the native AW algorithm (38.9% and 76.7%, respectively).

Ghosal et al. (2025) [37] and Weaver et al. (2025) [46] did not formally assess rhythm classification but reported that heart rate signals from AW were stable and potentially useful for future arrhythmia screening.

Overall, these findings indicate that while SW-derived ECG tracings can reliably support rhythm assessment when manually reviewed, current automated algorithms remain insufficient for independent diagnostic use in pediatric populations.

#### 3.2.2. Accuracy of Interval and Heart Rate Measurements

Strong correlation between SW-ECG and standard ECG intervals was established for HR and ECG intervals (PR, QT, QRS), yet systematic bias, particularly in QTc measurement, and lower accuracy for QRSd remain significant challenges.

Regarding heart rate and general intervals, correlations were strong across studies. Ernstsson et al. (2024) [36], utilizing the ScanWatch 1L, reported excellent agreement for HR (ICC ≥ 0.9), good agreement for PR and QT intervals (ICC = 0.75–0.89), but only moderate for QRSd (ICC = 0.5–0.74). The authors noted that the device significantly underestimated QRSd in 29% and QT interval in 60% of patients. Littell et al. (2022) [41] found strong correlations for HR (r = 0.96), QT (r = 0.9), PR (r = 0.79), and QRSd (r = 0.78), with agreement within ±20 ms observed in 66.3% of intervals. The superior performance of manual measurement was reported for the ScanWatch [36] and the AW [41] confirming the added value of clinician over-read.

Interestingly, Nash et al. (2024) [42] observed excellent heart rate accuracy during sinus rhythm (ICC 0.99), but severe underestimation during tachyarrhythmias (ICC 0.24–0.27), with devices unable to report HR > 210 bpm, representing a critical, clinically significant blind spot in the assessment of pediatric tachyarrhythmias.

Assessment of QTc revealed a systematic overestimation bias. Li et al. (2023) [40], in a study of long QT syndrome (LQTS) patients, found a strong positive correlation in QTc measurements across alternative leads (r = 0.9–0.93). Still, the Bland-Altman analysis revealed systematic QTc overestimation ranging from 9.37 ms to 21.47 ms across leads. This study demonstrated the SW-ECG’s strong ability to distinguish patients with and without QT prolongation, with AUC ranging from 0.87 to 0.99.

A different approach to improving diagnostic utility involves reconstructing multiple ECG lead recording from a single-lead device. Kurath-Koller et al. (2026) [30] evaluated such approach using alternative AW placements in pediatric cardiology patients. Although SW recordings demonstrated lower tracing quality (2/3 in a 3-point Likert scale vs. 3/3 with 12LECG and KM) and required longer acquisition time (median = 15 min vs. 4 min for 12LECG and 5 min for KM), moderate correlations were observed for PR interval and R peak time (r = 0.4 and 0.51, respectively) and almost-perfect correlation for QRS axis (r = 0.95).

Monitoring of continuous HR signals also demonstrated strong utility. Ghosal et al. (2025) [37] and Weaver et al. (2025) [46] extended these observations to continuous HR measurement with Apple Watch, Garmin, and Fitbit devices, showing moderate to strong agreement with reference Actiheart ECG and polysomnography, respectively. The AW generally provided the most accurate heart rate agreement with the compared device (r = 0.88–0.9 and r = 0.65, respectively).

The feasibility of SW-ECG acquisition has also been explored in very young populations. Paech et al. (2022) [43] evaluated the AW recordings in preterm neonates hospitalized in a neonatal intensive care unit. Two placements were investigated, with stronger correlation observed for wrist recordings compared with shoulder placement. Importantly, all tracings were interpretable after manual over-read, demonstrating the technical feasibility of SW-ECG acquisition even in extremely young pediatric patients.

#### 3.2.3. Detection of Other Conduction Abnormalities

The capacity of SW-ECGs to identify conduction system diseases is limited. Leroux et al. (2023) [39] reported the correct detection of atrioventricular blocks and SVTs, but under-recognition of LQTS and Wolff–Parkinson–White syndrome (WPW) at times. However, the device exhibited clinically relevant deficiencies in screening for specific channelopathies and accessory pathways: 4/5 patients with LQTS and 2/6 patients with WPW syndrome were missed.

Alternative electrode placements substantially mitigated these detection failures. Leroux et al. (2023) [39] demonstrated that placing the AW on the patient’s chest revealed the presence of a delta-wave in a patient with WPW, which was not visible on the standard wrist recording. This finding was echoed by Li et al. (2023) [40], who utilized alternative leads (lead II over the abdomen and lead V5 over the chest) in LQTS patients to improve QTc measurement accuracy and T-wave morphology assessment, with excellent intraobserver agreement (ICC = 1.0) in lead II.

Zahedivash et al. (2023) [47] showed that SW-ECGs led to new arrhythmia diagnoses in 71% of confirmed cases, most commonly indicating SVT (88%), VT (7%), or complete heart block (2.5%). Notably, rhythm abnormalities detected by the AW algorithm were not observed on ambulatory cardiac rhythm monitors in 29% of patients.

#### 3.2.4. Influence of Artifacts and Technical Factors

Technical limitations remain a key barrier in SW-ECG, particularly concerning body habitus and motion. Kobel et al. (2022) [38] found that higher body weight and BMI reduce P-wave (*p* = 0.029), QRS complex (*p* < 0.001), and T-wave (*p* < 0.025) amplitudes, thereby compromising signal quality (*p* = 0.029–<0.001). Motion artifacts and poor contact further degraded recordings, especially in younger or restless children. Modified lead placements, as proposed by Li et al. [40], substantially improved signal clarity. Ghosal et al. (2025) [37] also noted reduced HR accuracy during high-intensity activity and in children under 8 years due to motion artifacts and small wrist size. Weaver et al. (2025) [46] highlighted additional limitations during sleep in children with obesity (MAE +4.1 bpm) or obstructive sleep apnea (MAE +3.2 bpm). Similar technical challenges were reported by Kurath-Koller et al. (2026) [30], where attempts to obtain multi-lead SW recordings resulted in lower tracing quality and prolonged acquisition times, reflecting the practical difficulties of maintaining stable electrode contact during extended recordings.

#### 3.2.5. Patient Satisfaction

Roelle et al. (2022) [44] assessed the feasibility and acceptance of SW-ECGs in pediatric populations. 98% of parents and children reported that the devices were easy to use, and 87% expressed a willingness to continue using them. High acceptability and ease of implementation strongly support the potential for remote and home-based pediatric cardiac monitoring.

In summary, SW-ECGs are a reliable tool for pediatric cardiac monitoring, demonstrating high accuracy in HR and ECG interval assessment—particularly when tracings are manually reviewed, and alternative electrode placements are used. However, automated rhythm algorithms remain insufficient for stand-alone diagnostic use, and technical limitations related to motion artifacts, body size, and signal amplitude continue to constrain performance. Thus, SW-ECGs have value as screening and remote-monitoring tools, yet they should not be viewed as a replacement for standard 12LECG in definitive pediatric cardiac evaluation. Expert over-read remains essential to ensure diagnostic accuracy and clinical reliability.

### 3.3. Adhesive ECG Patches

Six studies evaluated the clinical utility of adhesive ECG patches in pediatric populations, comparing their performance with that of Holter monitors [48,49,50,51,52] or 12LECG [53] (Table 3). Some investigations also explored the feasibility of in-clinic versus mail-home self-application for long-term monitoring. Unlike previous wearable devices, the patches are more functionally comparable to Holter monitors than to a 12LECG, as they are designed primarily for extended monitoring rather than short-term evaluation.

The included pediatric cohorts were generally larger than those in other studies concerning portable ECG devices, with the largest trial, conducted by Bolourchi et al. (2015) [48], enrolled 3209 participants. Such a population size provided valuable insights into the diagnostic yield of those devices for arrhythmia detection, including SVT, VT, and sinus arrhythmias.

Adhesive ECG patch systems demonstrated consistently high recording quality and prolonged monitoring capability in pediatric cohorts. The proportion of interpretable recordings generally exceeded 92–98% [48,51], with relatively low artifact rates (2.8% [49] to 8.3% [51]). In specific infant populations, sternal placement can ensure even 100% interpretability of diagnostic tracings [53]. Clinically significant arrhythmias were detected in approximately 10% [52] to 17% [49] of monitored patients, depending on the clinical complexity of the cohort, with total arrhythmic yield higher for the patches compared to standard Holter monitors (10% vs. 9% [52]). A critical advantage of these systems is their extended monitoring capability. The proportion of arrhythmias detected beyond the standard 24-to-48-h monitoring window ranged from 44.1% [48] to 57% [52]. Moreover, ECG patches enable important clinical decisions—the findings from those devices can lead to facilitated discharge from cardiology follow-up reaching 46.8% of cases [50]. Patient adherence to the monitoring protocol was high, reaching 97.9% for 24-h monitoring time [48]. Interestingly, patches tend to fall off less often than traditional Holter leads (23% vs. 30% [49]), reflecting good tolerability of patch-based long-term monitoring in children.

#### 3.3.1. Arrhythmia Detection and Diagnostic Yield

Several studies demonstrated that extended monitoring increases the likelihood of detecting an arrhythmia compared with short-term recordings. Bolourchi et al. (2015) [48] conducted the first large-scale study evaluating the Zio XT Patch for pediatric arrhythmia detection. Asymptomatic arrhythmias were newly identified in 265 patients (8.3%) through continuous monitoring. The mean time to first detected arrhythmia was 2.7 ± 3.0 days, while the mean time to first symptom-triggered arrhythmia was 3.3 ± 3.3 days. Given that Holter monitoring is usually worn for up to 48 h, it is clinically significant that 44.1% of first-detected arrhythmias and 50.4% of symptom-triggered arrhythmias occurred beyond 48 h of monitoring. Similar findings were reported in other studies evaluating prolonged patch-based monitoring. Pradhan et al. (2019) [52] found that 57% of arrhythmias were detected after 24 h of Zio XT monitoring, further confirming its diagnostic advantage over short-term Holter use.

Available data suggest that patch-based systems provide diagnostic performance comparable to conventional Holter monitors. In a follow-up study, Bolourchi et al. (2020) [49] compared simultaneous 48-h recordings from Zio XT Patch and Holter monitors. Zio XT identified five SVT cases missed by Holter, whereas Holter detected only one missed by Zio. This difference, while not statistically significant, underscored Zio XT’s comparable diagnostic capability. Hitt et al. (2021) [50] not only reported non-inferiority of Zio XT in detection rate of serious arrhythmias requiring clinical intervention, but also found that patch monitoring more frequently provided results that allowed clinicians to safely discharge patients from follow-up (46.8% vs. 36.8%, *p* = 0.01). It highlights its potential role in ruling out clinically significant arrhythmias during symptomatic evaluation.

The adhesive patch monitoring may be beneficial not only for older children, but also for the youngest patients. Romme et al. (2021) [53] studied the use of a P-wave-centric Carnation Ambulatory Monitor (CAM) patch in infants weighing ≤10 kg with indications for ECG ambulatory monitoring. In this cohort, all recordings demonstrated clearly identifiable P waves, and monitoring results led to changes in clinical management in approximately 30% of patients (e.g., medication adjustments or discharge from cardiology care). Moreover, in one patient with tachycardia and unidentifiable P waves on 12LECG, patch recording demonstrated clear P waves, leading to a final diagnosis of atrial tachycardia.

In addition to comparable detection rates, the use of ECG patches may facilitate improved patient engagement during ambulatory monitoring. Khan et al. (2025) [51] found comparable rhythm detection rates during palpitations (51% with Zio XT versus 44% with Holter). Importantly, Zio XT users transmitted significantly more recordings (70% vs. 49%, *p* = 0.037), suggesting improved adherence and increased data acquisition during patch-based monitoring.

#### 3.3.2. Influence of Artifacts and Technical Factors

Artifact prevalence was low across studies, generally comparable to Holter performance. Bolourchi et al. (2015) [48] reported that 92.6% of total recording time was analyzable, with 7.4% excluded due to motion artifacts, particularly in younger patients. Despite this, most of those tracings were of diagnostic quality. Khan et al. (2025) [51] reported a 10% exclusion rate due to motion artifacts or suspected lead misplacement, predominantly in younger children, but still demonstrated the feasibility of unsupervised home use. Similarly, Pradhan et al. (2019) [52] reported 7% signal loss due to motion artifacts or poor lead contact, again more frequent in younger children.

Both Bolourchi et al. (2020) [49] and Pradhan et al. (2019) [52] found comparable artifact rates between the Zio XT Patch and traditional Holter monitor, indicating the device’s stable signal quality despite longer wear time. Proper patch placement and clear caregiver instructions were emphasized as key to minimizing artifacts.

#### 3.3.3. Patient and Clinician Satisfaction

Four studies assessed patient satisfaction and adherence. Bolourchi et al. (2015) [48] found that 75% of participants described the Zio XT Patch as comfortable, noting the absence of wires as a significant benefit, thereby increasing compliance. In their 2020 study, they also reported that 75% of patients preferred Zio XT over Holter due to comfort and ease of use. Additionally, 98% of patients were satisfied with Zio XT’s non-invasiveness. Pradhan et al. (2019) [52] confirmed these findings, with 90% of respondents describing Zio XT as easy to use. Khan et al. (2025) [51] similarly demonstrated higher patient satisfaction with Zio XT compared with Holter monitors (83% versus 58%).

Moreover, Romme et al. (2021) [53] described the positive clinician experience with the patch system. They underscored the advantages coming from low weight, lack of wires and simple configuration process. None of the recordings was prematurely discontinued and no skin reaction occurred.

Collectively, these results highlight that adhesive ECG patches provide high diagnostic yield, favorable user experience, and reliable signal quality, supporting their feasibility and acceptability for long-term, home-based pediatric cardiac monitoring.

## 4. Discussion

This review compared three main categories of ambulatory ECG technologies—handheld devices, SWs, and adhesive patches, focusing on their accuracy, usability, and suitability for pediatric populations [Table 4]. Each device type offers unique advantages and limitations. Handheld ECG serves as a convenient, event-based tool for rapid rhythm assessment during symptomatic episodes. Smartwatches, while less precise, represent the most accessible and widely adopted technology, enabling early, symptom-driven rhythm screening in older children and adolescents. Patch devices provide a continuous, long-term monitoring alternative to traditional Holter systems.

The primary outcome assessed across analyzed studies was the quality of tracings and the accuracy of HR and interval measurements obtained outside the hospital environment. When manually reviewed by trained professionals, KM and SW tracings demonstrated diagnostic quality comparable to standard 12LECG for assessing arrhythmias, QT prolongation, tachycardia, and atrioventricular blocks. However, automated rhythm algorithms—initially designed for adult populations—showed reduced accuracy in children, often yielding inconclusive or false-positive results. This limitation extends beyond the lack of pediatric-specific validation and reflects developmental physiology—higher baseline heart rates, shorter PR and QRS intervals, and the physiological respiratory sinus arrhythmia typical of infants and young children can create “algorithmic blind spots”. This is predisposing mobile devices to misclassify normal pediatric patterns as irregular rhythms. These factors underscore the necessity of expert over-read in all pediatric applications. In the context of interval assessment, particularly QTc, it is also important to note that Bazett correction becomes less reliable at the high HR common in children [54], meaning that wearable-obtained QTc values may support screening but cannot replace a 12-lead ECG for definitive channelopathy evaluation. Advances in artificial intelligence and machine learning are expected to enhance diagnostic performance in future software updates.

In addition to physiological considerations, technical constraints of wearable devices at very high heart rates must be acknowledged. Several smartwatch-based algorithms are known to lose accuracy or fail to classify rhythms above approximately 200–210 bpm, a range frequently exceeded in infant supraventricular tachycardia. As a result, devices may fail precisely when diagnostic capture is most clinically valuable, further supporting the need for consistent manual evaluation of pediatric tracings.

Direct comparisons between mobile ECG technologies and conventional ambulatory recorders showed that diagnostic yield was similar, with mobile devices generating more tracings and fewer data transmission issues. Patients consistently reported greater comfort and ease of use with mobile devices, attributing this to the absence of adhesive electrodes, compact form factor, and user-friendly mobile applications. Such features translated into higher adherence and overall satisfaction, suggesting that mobile ECGs may improve long-term engagement in pediatric cardiac monitoring.

An important consideration for future development is the inclusion of younger pediatric patients, particularly those under three years of age, who remain underrepresented in existing studies [55]. Most available research focus on school-age children and adolescents, leaving a significant evidence gap for the younger children. In this group, KM presents practical challenges, as recordings require continuous finger contact for approximately 30 s—an unrealistic expectation for infants and toddlers. Expanding the applicability of mobile ECGs in this age range will likely need hardware redesign or modified recording methods, such as alternative electrode placement, shorter automatic recordings, or integration with wearable sensors. These adaptations could substantially enhance accessibility and diagnostic capability in early childhood populations.

Across all device types, a significant benefit lies in the extended monitoring duration, which increases the likelihood of capturing both symptomatic and asymptomatic arrhythmias that short-term Holter recordings may miss. Unlike event recorders, which depend on user activation, or Holters, which are limited to 24–72 h of recording, SW-ECGs and adhesive patches enable prolonged or continuous rhythm tracking. This provides a more comprehensive view of a patient’s cardiac rhythm over time, with particular value in detecting intermittent arrhythmias.

The overall quality of ECG recordings was closely linked to user experience and technical factors. Artifacts were more frequent among younger children and those with higher BMI, due to motion, limited cooperation, or suboptimal skin contact. Studies consistently emphasized the importance of adequate patient and caregiver education, as proper device use and electrode placement significantly reduced artifacts and improved diagnostic reliability. Even imperfect recordings, however, were often sufficient for clinical interpretation, reaffirming the practical feasibility of these devices in pediatric care.

There are several limitations regarding the evidence obtained during the analysis. One of them is the limited availability of data for very young children, which remains an important gap in the current literature. Furthermore, many studies—especially regarding KM and SW—were conducted on relatively small population sizes. The research also varies in terms of the addressed endpoints, at times limiting the potential for direct head-to-head analysis of the results across the publications. An additional consideration is the regulatory and ethical context, as most of the devices evaluated in the included studies were used off-label in pediatric populations, which may influence both study design and clinical applicability.

The review process itself also had significant limitations that should be addressed. The primary limitation was the inability to perform a formal meta-analysis due to high methodological and technical heterogeneity across studies, as different devices and outcome measures were utilized.

## 5. Conclusions

Mobile and wearable ECG devices demonstrate promising clinical utility in pediatric populations. Handheld devices and SWs produce tracings of comparable quality to standard 12LECG when manually reviewed, while adhesive patches offer diagnostic performance comparable with conventional Holter monitoring. Automated algorithms currently lack the reliability required for independent pediatric diagnosis, but with professional interpretation, these recordings provide accurate and clinically actionable data. Moreover, AI-assisted algorithms offer a promising approach to automated tracing interpretation.

To optimize the use of such devices in children, especially the youngest ones, algorithms must be refined to account for pediatric-specific ECG morphology and rhythm variability. Further research is essential to validate device performance in younger cohorts and to explore innovations in electrode design and sensor integration.

In summary, mobile ECG technologies hold substantial value as screening and remote-monitoring tools, improving accessibility and patient engagement. However, they should be viewed as complementary rather than replacement modalities for standard 12LECG in definitive pediatric cardiac evaluation.

## Figures and Tables

**Table 1:** Characteristics and key findings of studies investigating handheld ECG devices use in pediatric ambulatory ECG monitoring.

Study Number	Author	Device	Enrollment Period	Indications for ECG Monitoring	Population Size	Use of a Control Device	Age IN Years (Mean ± SD)	Outcomes
1	Al Riyami et al. (2025) [23]	KM1L	N/A	Palpitations	100	CardioCall (conventional event recorder)	14.1 ± 3.4 -> MDG 14.3 ± 3.1 -> SDG	* More patients from KM transmitted a recording compared to the conventional group (70% vs. 49%, *p* = 0.037) * Patient satisfaction was higher for KM users (83% vs. 58%)
2	Al-mousily et al. (2021) [24]	KM1L	June 2018–August 2019	Palpitations, chest pain, syncope	126	CorDigital MicroER (conventional event recorder)	15 ± 9 -> MDG 13 ± 8 -> SDG	* Survey showed that overall KM was easy to use with high patient satisfaction rate
3	Dahlqvist et al. (2014) [25]	Zenicor-ECG	N/A	Patients with Fontan circulation	27	Holter monitors	9.5 (range 2.7–16.5)	* Perfect correlation (r = 1, *p* < 0.001) for interbeat intervals compared to simultaneous Holter * Handheld ECG detected SVT missed by previous 24-h Holter and 12LECG * 100% adherence over 14 days
4	Ferdman et al. (2015) [26]	KM1L	June 2013–February 2014	Patients undergoing ablation for SVT	37	Standard 12L ECG	13.7 ± 2.8	* Rhythm identification accuracy—59–73% * Comparability to standard ECG—51–86%
5	Girvin et al. (2023) [27]	KM6L	June 2021–November 2021	Patients with congenital/ structural heart disease	141	Standard 15L ECG	12.3 ± 4.4	* Rhythm identification accuracy—Sensitivity for prolonged QTc (>460 ms) = 54%, specificity = 95%. For prolonged QRS ≥ 120 ms: sensitivity = 80%, specificity = 97%. * Comparability to standard ECG—QTc mean difference –0.6 ms (95% CI −48 to +47 ms), 83% within 30 ms, 64% within 20 ms. >QRS mean difference −1.3 ms (95% CI −23 to +21 ms), 95% within 20 ms. >Axis identical in 84% of cases
6	Gropler et al. (2018) [28]	KM1L	N/A	Pediatric patients (20 (67%) with CHD and/or conduction abnormalities)	30	Standard 12L ECG	8.2	* Comparability to standard ECG—Very strong correlation for PR, QRS, QTc (r > 0.8) + for automated-vs-measured KM HR and automated KM-vs-EP calculated 12L ECG HR
7	Karacan et al. (2019) [29]	KM1L	N/A	Pediatric patients	285	Standard 12L ECG	9.8 ± 4.9	* Comparability to standard ECG—Very strong correlation for mean HR (r = 0.92) and mean QT (r = 0.83), moderate for QTc (r = 0.57)
8	Kurath-Koller et al. (2026) [30]	KM6L	N/A	Pediatric patients without known cardiac conditions	30	Standard 12L ECG	11.5 (range 4–17)	* Very strong correlation for QRS axis and R volatage (r = 0.8–1.0), strong for T wave voltage and QT interval (r = 0.6–0.79), moderate for HR (0.4–0.59) * High interobserver agreement for tracing quality * Mean quality score for KM was similar to 12L ECG (2.7 ± 0.5 vs. 2.8 ± 0.4 in a 3-point Likert scale)
9	Lawley et al. (2024) [31]	KM6L	N/A	Patients with Inherited Heart Conditions [HCM—22 (34.4%) LQTS—22 (34.4%)]	64	Standard 12L ECG	12 ± 3	* Comparability to standard ECG—ICC strong positive for QRSd and QTc, moderate for HR Substantial agreement for abnormal QTc (kappa 0.714, *p* < 0.001)
10	Macinnes et al. (2019) [32]	KM1L	24 months	Palpitations	180	N/A	11 (median)	* 98% of patients from the KM group sent at least one recording compared to 69% from the conventional group (*p* < 0.05) * Uninterpretable due to artifacts: 8.3% in KM vs. 8% in conventional
11	Miller et al. (2024) [33]	KM6L	N/A	Patients scheduled for ablation	28	Standard 12L ECG	13.2 ± 4	* 100% reported that KM was easy to use * 92% reported that KM app was easy to use * 96% felt comfortable with using the device without a health-care professional
12	Nguyen et al. (2015) [34]	KM1L	September 2013–September 2014	Documented paroxysmal arrhythmia (SVT—57% AF—11% Ectopic AT—6% AT—3% VT—23%)	35	N/A	12 (median)	* 96% of transmitted tracings were of diagnostic quality * 4% of tracings were uninterpretable due to too much noise/motion artifact

* Marks key outcomes extracted from the original study. Abbreviations: AW, Apple Watch; KM1L, Single-lead KardiaMobile; KM6L, Six-lead KardiaMobile; 12LECG, 12-lead ECG; MDG, Mobile Device Group; SDG, Standard Device Group; SVT, Supraventricular Tachycardia; QTc, corrected QT interval; ECG, Electrocardiogram; CI, Confidence Interval; CHD, Congenital Heart Defect; HR, Heart Rate; HCM, Hypertrophic Cardiomyopathy; LQTS, Long QT Syndrome; QRSd, QRS duration; AF, Atrial Fibrillation; AT, Atrial Tachycardia; VT, Ventricular Tachycardia.

**Table 2:** Characteristics and key findings of studies evaluating smartwatch-derived ECGs in pediatric cardiac monitoring.

Study Number	Author	Device	Enrollment Period	Indications for ECG Monitoring	Population Size	Use of Control Device	Age in Years [Mean ± SD (Range)]	Outcomes
1	Ernstsson et al. (2024) [36]	Withings ScanWatch 1L	September 2023–October 2023	Routine outpatient pediatric cardiology patients	100	N/A	12.9 (median)	* Automated and manual measurements compared between 12LECG and smartwatch ECG
2	Ghosal et al. (2025) [37]	Apple Watch Series 7, Garmin Vivoactive 4, Fitbit Sense	May 2022–March 2024	Children 5–12 y/o	432	N/A	9.3 ± 2.1	* HR accuracy: Apple r = 0.88–0.9; Garmin r = 0.82–0.95; Fitbit r = 0.6–0.82
3	Kobel et al. (2022) [38]	Apple Watch series 3	N/A	Routine pediatric cardiology patients	215	N/A	8.05 ± 4.33	* Sinus rhythm identified in 55.7%, 37.9% classified as inconclusive, 94.9% of recordings were evaluable, comparable to standard ECG.
4	Kurath-Koller et al. (2026) [30]	Apple Watch series 6	N/A	Pediatric cardiology patients without known cardiac conditions	30	12LECG	11.5 (range 4–17)	* Alternative device placements were implemented to obtain a 12-lead recording * Lower tracing quality (2/3 in Likert scale) and longer acquisition time (median = 15 min) compared to 12LECG (3/3 and 4 min) and KM recordings (3/3 and 5 min) * Low-to-moderate correlation for all ECG intervals and HR
5	Leroux et al. (2023) [39]	Apple Watch series 5	N/A	Normal or abnormal ECG	110	N/A	4.9 ± 4	* Sensitivity 86% (95% CI 70–95), specificity 100% (95% CI 95–100) for detection of abnormal ECG.
6	Li et al. (2023) [40]	Apple Watch series 6	April 2022–October 2022	Congenital LQTS and other arrhythmias	136	N/A	13.5 ± 3	* QTc values: strong correlation (r = 0.91, *p* < 0.001) between AW and 12LECG
7	Littell et al. (2022) [41]	Apple Watch series 6	5 weeks	Pediatric patients who were ordered an ECG	84	N/A	7.2 ± (0–18)	* Manual physician interpretation of AW6 ECG: 100% agreement with 12LECG for rhythm. AW6 automated algorithm: 65.6% accurate.
8	Nash et al. (2024) [42]	Apple Watch series 5	1 year	Pediatric patients undergoing routine ECG evaluation	80	N/A	13 (median)	* Sensitivity 89–96%, specificity 78–87% for identifying non-sinus rhythm.
9	Paech et al. (2022) [43]	Apple Watch series 4	N/A	Pre-term neonates hospitalized at the neonatal ICU	50	12LECG	31 ± 4	* Two AW placements were investigated—wrist placement and shoulder placement * The correlation was stronger between 12LECG and wrist AW placement compared to shoulder placement * 100% interpretability on manual over-read
10	Roelle et al. (2022) [44]	Apple Watch series 6, KM1L, Coala monitor	2020–2021	Pediatric patients scheduled for a telehealth visit	30	N/A	11.6 ± (0–20)	* Providers rated ECG quality as high (Apple Watch 93%, Coala 86%, Kardia 62%)
11	Teich et al. (2023) [45]	Apple Watch series 6	March 2022–October 2022	Pediatric patients with or without CHD	48	N/A	9.9 ± (0–18)	* Apple algorithm: sensitivity 38.9%, specificity 76.7% for sinus rhythm vs. pathology.
12	Weaver et al. (2025) [46]	Apple Watch Series 7, Garmin Vivoactive 4, Fitbit Sense	March 2022–March 2023	Pediatric patients with suspected sleep disruption	82	N/A	8.4 ± 2.2	* HR—AWS7: r = 0.65, Lin’s CCC = 0.61, MAE = 6.4 bpm, MAPE = 7.3%; GT9X: poor (r = −0.12, CCC = −0.11, MAE 16.8 bpm, MAPE 20%) * Obese children had higher error (MAE +4.1 bpm vs. normal weight)
13	Zahedivash et al. (2023) [47]	Apple Watch series 4 and up	2018–2022	Patients with suspected arrhythmias	41	N/A	13.8 ± 3.2	* 71% of patients—new arrhythmia diagnosis thanks to Apple Watch findings

* Marks key outcomes extracted from the original study. Abbreviations: SD, standard deviation; 12LECG, 12 Lead Electrocardiogram; y/o, years old; KM, KardiaMobile; LQTS, long QT syndrome; QTc, corrected QT interval; AW6, Apple Watch series 6; ICU, intensive care unit; AW, Apple Watch; HR, heart rate; KM1L, KardiaMobile 1 Lead; CHD, Congenital Heart Disease; HR, Heart Rate; AWS7, Apple Watch series 7; GT9X, ActiGraph GT9X Link; MAE, mean absolute error; MAPE, mean absolute percent error; REM, rapid eye movement.

**Table 3:** Summary of study characteristics and principal findings on the use of adhesive ECG patches in pediatric ambulatory rhythm monitoring.

Study Number	Author	Device	Enrollment Period	Indications for ECG Monitoring	Population Size	Use of Control Device	Age in Years (Mean ± SD)	Outcomes
1	Bolourchi et al. (2015) [48]	Zio XT patch	January 2011–December 2014	Palpitations, syncope, unsepcified tachycardia, paroxysmal SVT, chest pain	3209	N/A	12.5 ± 4.4	* Wear-time compliance lowered significantly with every extra day of wearing it * Asymptomatic arrhythmias detected de novo in 265 patients (8.3%) by continuous recording
2	Bolourchi et al. (2020) [49]	Zio XT patch	October 2017–May 2019	Consecutive patients scheduled for ambulatory ECG monitoring	200	Holter monitor	13.5 (median)	* Median rhythm artifact time lower for Zio compared to Holter (2.8% (1.1–8.6) vs. 5.6% (2.4–15.7), *p* < 0.001) * 75% of patients preferred Zio > Holter (*p* < 0.001) according to the survey results
3	Hitt et al. (2021) [50]	Zio XT patch	May 2015–February 2017	Patients with indication for prolonged ECG monitoring	483	Event/Holter monitors	Patch group: 11.1 ± 4.9 Control device group: 9.4 ± 5.8	* Arrhythmia detection rates leading to interventions were similar for both groups (6.2% for Zio vs. 7.7% for control group, *p* = 0.55) * Zio patch recordings allowed patient discharge from cardiology follow-up more often than control devices (46.8% vs. 36.8%, *p* = 0.01)
4	Khan et al. (2025) [51]	Zio XT patch	January 2018–June 2021	Patients scheduled for ambulatory visits	294	N/A	In-clinic 12.8 Mail-home 11.1	* Artifact decreased with increasing patient age (~0.34% reduction per year, *p* < 0.05)
5	Pradhan et al. (2019) [52]	Zio XT patch	October 2014–February 2016	Patients scheduled for ambulatory ECG monitoring with suspected or documented arrhythmia (SVT or VT)	905	Holter monitor	Zio Patch—12.7 Holter—4.9 (median)	* Zio XT detected 57% of arrhythmias after 24 h, while Holter detected all arrhythmias within 24 h
6	Romme et al. (2021) [53]	CAM patch	N/A	Patients with body weight ≤ 10 kg who underwent 48-h CAM patch ambulatory monitoring	25	12LECG	4.2 ± 5 [months]	* P wave morphology in patch recordings resembled the corresponding 12LECG in 77% of cases * In one patient, CAM patch recording unveiled P waves unrecognizable on 12LECG, leading to a diagnosis of atrial tachycardia * Positive clinician experience with the patch

* Marks key outcomes extracted from the original study. Abbreviations: SVT, Supraventricular Tachycardia; VT, ventricular tachycardia; ECG, Electrocardiogram; CAM, Carnation Ambulatory Monitor; 12LECG, 12-lead ECG.

**Table 4:** Study characteristics across three categories of ambulatory rhythm-monitoring technologies: handheld ECG devices, smartwatches, and adhesive ECG patches.

Parameter	Handheld ECG Devices	Smartwatches	Adhesive ECG Patches
Number of included studies	12	13	6
Population size (range)	27–285	30–432	26–3209
Interpretable/ Diagnostic-quality recordings	90–96% [32,34]	94.9–100% [38,43]	91.7–95% analyzable time [48,51]
Diagnostic performance/clinical yield	Arrhythmia mechanism differentiation accuracy 59–76% [26]	Sensitivity 38.9–96%, specificity 76.7–100% for rhythm classification [39,42,45]	Asymptomatic arrhythmias detected de novo in 265 patients (8.3%) [48] Arrhythmia detection leading to clinical intervention 6.2–7.7% [50]
Heart rate correlation with reference ECG	r = 0.44–0.92 [29,30]	r = 0.65–0.96 [37,41,46]	Average of 85 ± 15 (Zio) versus 86 ± 15 (Holter) beats per minute [49]
QT/QTc agreement with reference ECG	r = 0.57–0.83, sensitivity 54–80%, specificity 95–96% [27,28,29]	r = 0.90–0.93 with systematic QTc overestimation 9–21 ms [40]	N/A
Artifact rate	7–35% depending on configuration and age [27,29,31,32]	0–19% [37,38]	2.8–8.3% [48,49,51]
Patient usability/ satisfaction	Satisfaction 89% vs. 38% conventional group [32] Satisfaction 83% vs. 58% convectional group [23]	98% easy to use; 87% willing to continue use [44]	75% preferred patch vs. Holter [49]
Typical monitoring duration	Symptom-triggered recordings	Short ECG recordings	7.8 ± 4.4 days [48] 48.2 h (IQR: 45.8 to 50.2) of wear-time

## Data Availability

This study is based solely on previously published data. No new datasets were created, and all sources included in the review are publicly available through the databases listed in Section 2.

## Supplementary Materials

The following supporting information can be downloaded at: https://www.mdpi.com/article/10.3390/jcm15082883/s1, Table S1: PRISMA 2020 Checklist. Table S2: Risk of bias assessment of diagnostic accuracy studies with the QUADAS-2 instrument; Table S3: Risk of bias assessment of cohort studies with the Newcastle-Ottawa Scale for assessing the quality of nonrandomized studies; Table S4: Risk of bias assessment with revised Cochrane risk-of-bias tool for randomized trials (RoB 2).

## Abbreviations

The following abbreviations are used in this manuscript:

12LECG	12-lead ECG
AF	atrial fibrillation
AT	atrial tachycardia
AVRT	atrioventricular reentrant tachycardia
AVNRT	atrioventricular nodal reentrant tachycardia
AW	Apple Watch
CHD	congenital heart disease
ECG	electrocardiogram
ESC	European Society of Cardiology
HR	heart rate
KM	KardiaMobile
KM1L	Single-lead KardiaMobile
KM6L	Six-lead KardiaMobile
LQTS	long-QT syndrome
QRSd	QRS duration
QTc	corrected QT interval
SW	smartwatch
SW-ECG	smartwatch-derived electrocardiograms
VT	ventricular tachycardia
SVT	supraventricular tachycardia
WPW	Wolff-Parkinson-White syndrome
