# Supplementary material for: Wearable and Portable Electrocardiographic Devices as Modern Cardiac Telemetry Solutions in Pediatrics: A Systematic Review
Source: J Clin Med. 2026 Apr 10;15(8):2883. doi: 10.3390/jcm15082883 (PMC13115905; doi:10.3390/jcm15082883)
Supplement: Supplementary file 1 [file jcm-15-02883-s001.zip › jcm-4186530-supplementary.pdf]

Table S1. PRISMA 2020 Checklist.

Section and Topic	Item #	Checklist item	Location where item is reported
**TITLE**			
Title	1	Identify the report as a systematic review.	Title
**ABSTRACT**			
Abstract	2	See the PRISMA 2020 for Abstracts checklist.	Lines 11-37
**INTRODUCTION**			
Rationale	3	Describe the rationale for the review in the context of existing knowledge.	Lines 43-118
Objectives	4	Provide an explicit statement of the objective(s) or question(s) the review addresses.	Lines 122-127
**METHODS**			
Eligibility criteria	5	Specify the inclusion and exclusion criteria for the review and how studies were grouped for the syntheses.	Lines 135-138; Lines 147-149
Information sources	6	Specify all databases, registers, websites, organisations, reference lists and other sources searched or consulted to identify studies. Specify the date when each source was last searched or consulted.	Lines 126-128
Search strategy	7	Present the full search strategies for all databases, registers and websites, including any filters and limits used.	Lines 138-140
Selection process	8	Specify the methods used to decide whether a study met the inclusion criteria of the review, including how many reviewers screened each record and each report retrieved, whether they worked independently, and if applicable, details of automation tools used in the process.	Lines 140-145
Data collection process	9	Specify the methods used to collect data from reports, including how many reviewers collected data from each report, whether they worked independently, any processes for obtaining or confirming data from study investigators, and if applicable, details of automation tools used in the process.	Lines 140-145
Data items	10a	List and define all outcomes for which data were sought. Specify whether all results that were compatible with each outcome domain in each study were sought (e.g. for all measures, time points, analyses), and if not, the methods used to decide which results to collect.	Lines 147-149
	10b	List and define all other variables for which data were sought (e.g. participant and intervention characteristics, funding sources). Describe any assumptions made about any missing or unclear information.	Not included
Study risk of bias assessment	11	Specify the methods used to assess risk of bias in the included studies, including details of the tool(s) used, how many reviewers assessed each study and whether they worked independently, and if applicable, details of automation tools used in the process.	Lines 159-168
Effect measures	12	Specify for each outcome the effect measure(s) (e.g. risk ratio, mean difference) used in the synthesis or presentation of results.	No meta-analysis was performed
Synthesis methods	13a	Describe the processes used to decide which studies were eligible for each synthesis (e.g. tabulating the study intervention characteristics and comparing against the planned groups for each synthesis (item #5)).	No meta-analysis was performed
	13b	Describe any methods required to prepare the data for presentation or synthesis, such as handling of missing summary statistics, or data conversions.	No meta-analysis was performed
	13c	Describe any methods used to tabulate or visually display results of individual studies and syntheses.	No meta-analysis was performed
	13d	Describe any methods used to synthesize results and provide a rationale for the choice(s). If meta-analysis was performed, describe the model(s), method(s) to identify the presence and extent of statistical heterogeneity, and	No meta-analysis was performed

Section and Topic	Item #	Checklist item	Location where item is reported
		software package(s) used.	
	13e	Describe any methods used to explore possible causes of heterogeneity among study results (e.g. subgroup analysis, meta-regression).	No meta-analysis was performed
	13f	Describe any sensitivity analyses conducted to assess robustness of the synthesized results.	No meta-analysis was performed
Reporting bias assessment	14	Describe any methods used to assess risk of bias due to missing results in a synthesis (arising from reporting biases).	No meta-analysis was performed
Certainty assessment	15	Describe any methods used to assess certainty (or confidence) in the body of evidence for an outcome.	No meta-analysis was performed
**RESULTS**			
Study selection	16a	Describe the results of the search and selection process, from the number of records identified in the search to the number of studies included in the review, ideally using a flow diagram.	Lines 150-156, Figure 1.
	16b	Cite studies that might appear to meet the inclusion criteria, but which were excluded, and explain why they were excluded.	All records have been retrieved.
Study characteristics	17	Cite each included study and present its characteristics.	Table 1,<br>Table 2,<br>Table 3.
Risk of bias in studies	18	Present assessments of risk of bias for each included study.	Table S2, Table S3, Table S4<br>Risk of bias scores presented as a mean of two independent researchers' assessments.<br>QUADAS-2:<br>Al-Mousily et al. (2021)<br>Bolourchi et al. (2020)<br>Erntsson et al. (2024)<br>Ferdman et al. (2015)<br>Girvin et al. (2023)<br>Gropler et al. (2018)<br>Kurath-Koller et al. (2026)<br>Lawley et al. (2024)<br>Leroux et al. (2023)<br>Li et al. (2023)<br>Littell et al. (2022)<br>Nash et al. (2024)

Section and Topic	Item #	Checklist item	Location where item is reported
			Paech et al. (2022)<br>Pradhan et al. (2019)<br>Newcastle-Ottawa Scale:<br>Bolourchi et al. (2015)<br>Dahlqvist et al. (2014)<br>Ghosal et al. (2025)<br>Hitt et al. (2021)<br>Khan et al. (2025)<br>Kobel et al. (2022)<br>Karacan et al. (2019)<br>MacInnes et al. (2019)<br>Miller et al. (2024)<br>Nguyen et al. (2015)<br>Roelle et al. (2022)<br>Teich et al. (2023)<br>Weaver et al. (2025)<br>Zahedivash et al. (2023)<br><br>RoB 2:<br>Al Riyami et al. (2025)
Results of individual studies	19	For all outcomes, present, for each study: (a) summary statistics for each group (where appropriate) and (b) an effect estimate and its precision (e.g. confidence/credible interval), ideally using structured tables or plots.	Table 1, Table 2, Table 3<br>Results section
Results of syntheses	20a	For each synthesis, briefly summarise the characteristics and risk of bias among contributing studies.	Lines 159-168, Table S2, Supplementary material<br>Lines 291-297, Table S3, Supplementary material<br>Lines 405-416, Table S4, Supplementary material
	20b	Present results of all statistical syntheses conducted. If meta-analysis was done, present for each the summary estimate and its precision (e.g. confidence/credible interval) and measures of statistical heterogeneity. If comparing groups, describe the direction of the effect.	No meta-analysis was performed
	20c	Present results of all investigations of possible causes of heterogeneity among study results.	No meta-analysis was performed
	20d	Present results of all sensitivity analyses conducted to assess the robustness of the synthesized results.	No meta-analysis was performed
Reporting biases	21	Present assessments of risk of bias due to missing results	No meta-analysis

Section and Topic	Item #	Checklist item	Location where item is reported
		(arising from reporting biases) for each synthesis assessed.	was performed
Certainty of evidence	22	Present assessments of certainty (or confidence) in the body of evidence for each outcome assessed.	No meta-analysis was performed
**DISCUSSION**			
Discussion	23a	Provide a general interpretation of the results in the context of other evidence.	Lines 623-640, Table 4
	23b	Discuss any limitations of the evidence included in the review.	Lines 678-686
	23c	Discuss any limitations of the review processes used.	Lines 687-690
	23d	Discuss implications of the results for practice, policy, and future research.	Lines 654-663
**OTHER INFORMATION**			
Registration and protocol	24a	Provide registration information for the review, including register name and registration number, or state that the review was not registered.	Lines 131-132
	24b	Indicate where the review protocol can be accessed, or state that a protocol was not prepared.	Lines 131-132
	24c	Describe and explain any amendments to information provided at registration or in the protocol.	None made
Support	25	Describe sources of financial or non-financial support for the review, and the role of the funders or sponsors in the review.	Line 722
Competing interests	26	Declare any competing interests of review authors.	Line 728
Availability of data, code and other materials	27	Report which of the following are publicly available and where they can be found: template data collection forms; data extracted from included studies; data used for all analyses; analytic code; any other materials used in the review.	Lines 708-715

Table S2. Risk of bias assessment of diagnostic accuracy studies with the QUADAS-2 instrument. - High, ? Unclear, + Low.

**Author et al. (year)**	**Patient selection**		**Index test**		**Reference standard**		**Flow and timing**
	Risk of bias	Applicability concern	Risk of bias	Applicability concern	Risk of bias	Applicability concern	Risk of bias
Al-Mousily et al. (2021)	?	+	?	+	?	+	-
Bolourchi et al. (2020)	+	+	+	+	+	+	?
Ernstsson et al. (2024)	+	+	?	+	+	+	+
Ferdman et al. (2015)	+	?	+	?	+	+	?
Girvin et al. (2023)	+	+	?	+	+	+	?
Gropler et al. (2018)	+	+	-	+	+	+	-
Kurath-Koller et al. (2026)	?	?	+	?	+	+	?
Lawley et al. (2024)	?	?	+	+	+	+	+
Leroux et al. (2023)	-	+	+	+	+	+	+
Li et al. (2023)	?	+	+	+	+	+	+
Littell et al. (2022)	?	+	+	+	+	+	-
Nash et al. (2024)	-	+	+	+	+	+	+
Paech et al. (2022)	+	?	+	+	+	+	+
Pradhan et al. (2019)	?	-	?	+	?	+	?

Table S3. Risk of bias assessment of cohort studies with the Newcastle-Ottawa Scale for assessing the quality of nonrandomized studies.

Author et al. (year)	Selection				Comparability	Outcome			Total (maximum 9 points)
	Representativeness of exposed cohort	Selection of non-exposed cohort	Ascertainment of exposure	Outcome not present at start		Assessment of outcome	Follow-up long enough	Adequacy of follow-up	
Bolourchi et al. (2015)	★		★	★	★	★	★	★	**7**
Dahlqvist et al. (2014)	★		★	★	★★	★	★	★	**8**
Ghosal et al. (2025)	★	★	★	★	★★	★		★	**8**
Hitt et al. (2021)	★		★	★	★	★	★	★	**7**
Khan et al. (2025)	★	★	★	★	★	★	★	★	**8**
Kobel et al. (2022)	★	★	★	★	★★	★		★	**8**
Karacan et al. (2019)	★	★	★	★	★★	★		★	**8**
Macinnes et al. (2019)	★	★	★	★	★	★	★	★	**8**

Miller et al. (2024)	★		★	★		★		★	**5**
Nguyen et al. (2015)	★		★	★	★	★	★	★	**7**
Roelle et al. (2022)	★		★	★		★		★	**5**
Teich et al. (2023)	★	★	★	★	★★	★		★	**8**
Weaver et al. (2025)	★	★	★	★	★★	★		★	**8**
Zahedivash et al. (2023)	★		★	★		★	★	★	**6**

Table S4. Risk of bias assessment with revised Cochrane risk-of-bias tool for randomized trials (RoB 2).

**Author et al. (year)**	**Randomization process**	**Deviations from intended interventions**	**Missing outcome data**	**Measurement of the outcome**	**Selection of the reported result**	**Overall Bias**
Al Riyami et al. (2025)	Low risk	Low risk	Low risk	Low risk	Some concerns	Low risk
